# Bat rabies surveillance in France: first report of unusual mortality among serotine bats

**DOI:** 10.1186/s12917-017-1303-1

**Published:** 2017-12-13

**Authors:** Evelyne Picard-Meyer, Alexandre Servat, Marine Wasniewski, Matthieu Gaillard, Christophe Borel, Florence Cliquet

**Affiliations:** 1 ANSES Nancy Laboratory for Rabies and Wildlife, European Union Reference Laboratory for Rabies, WHO Collaborating Centre for Research and Management in Zoonoses Control, OIE Reference Laboratory for Rabies, European Union Reference Institute for Rabies Serology, Technopôle agricole et vétérinaire de Pixérécourt, CS 40009, 54220 Malzéville, France; 2 Néomys association, Centre Ariane, 240 rue de Cumène, 54230 Neuves-Maisons, France; 3 CPEPESC-Lorraine, Centre Ariane, 240 rue de Cumène, 54230 Neuves-Maisons, France

**Keywords:** Rabies, EBLV-1, Bat, Mortality

## Abstract

**Background:**

Rabies is a fatal viral encephalitic disease that is caused by lyssaviruses which can affect all mammals, including human and bats. In Europe, bat rabies cases are attributed to five different lyssavirus species, the majority of rabid bats being attributed to European bat 1 lyssavirus (EBLV-1), circulating mainly in serotine bats (*Eptesicus serotinus*). In France, rabies in bats is under surveillance since 1989, with 77 positive cases reported between 1989 and 2016.

**Case presentation:**

In the frame of the bat rabies surveillance, an unusual mortality of serotine bats was reported in 2009 in a village in North-East France. Six juvenile bats from an *E. serotinus* maternity colony counting ~200 individuals were found to be infected with EBLV-1. The active surveillance of the colony by capture sessions of bats from July to September 2009 showed a high detection rate of neutralising EBLV-1 antibodies (≈ 50%) in the colony. Moreover, one out of 111 animals tested was found to shed viable virus in saliva, while lyssavirus RNA was detected by RT-PCR for five individuals.

**Conclusion:**

This study demonstrated that the lyssavirus infection in the serotine maternity colony was followed by a high rate of bat rabies immunity after circulation of the virus in the colony. The ratio of seropositive bats is probably indicative of an efficient virus transmission coupled to a rapid circulation of EBLV-1 in the colony.

**Electronic supplementary material:**

The online version of this article (10.1186/s12917-017-1303-1) contains supplementary material, which is available to authorized users.

## Background

Rabies is an ancient viral zoonotic disease caused by negative-strand RNA viruses of the genus lyssavirus, family *Rhabdoviridae*, known to affect the central nervous system of mammals including humans. There are currently 14 recognised species in the lyssavirus genus [1] the prototype virus species being rabies lyssavirus (RABV), which is found in a plethora of terrestrial mammals worldwide and in bats in the Americas. With the exception of two species -Mokola lyssavirus and Ikoma lyssavirus- all lyssaviruses have been isolated from bats [2].

In Europe, bat rabies is caused by five different lyssavirus species: European bat 1 lyssavirus (EBLV-1), European bat 2 lyssavirus (EBLV-2), Bokeloh bat lyssavirus (BBLV), West Caucasian bat lyssavirus (WCBV) and Lleida bat lyssavirus (LLEBV), a putative species detected in Spain in *Miniopterus schreibersii* [3]. While EBLV-1 has been mainly associated with serotine bats and reported across much of Europe [4], EBLV-2 has been isolated from Myotis bats (*Myotis dasycneme* and *Myotis daubentonii*). BBLV, recently reported in France and Germany, is mainly associated with *Myotis nattereri* [5, 6], while WCBV has only been isolated once in *Miniopterus schreibersii* [7]. In France, the bat rabies surveillance scheme -involving in particular veterinary services and the national bat conservation network (SFEPM) [8]- reported 45 serotine bats infected with EBLV-1 between 1989 and 2009. In 2009, an unusual case of mortality of bats was reported in the Moselle department located in North-East France, in the frame of the bat rabies surveillance. Neither lyssavirus infection was found in bats originating from the Moselle department between 1989 and 2009, nor unusual mortality except classic mortality of juveniles. We report here an unusual case of mortality in a colony of bats in France, coupled to the presence of EBLV-1 in the colony, as well as an high proportion of seropositive individuals in that colony.

## Case presentation

**Fig. 1:**
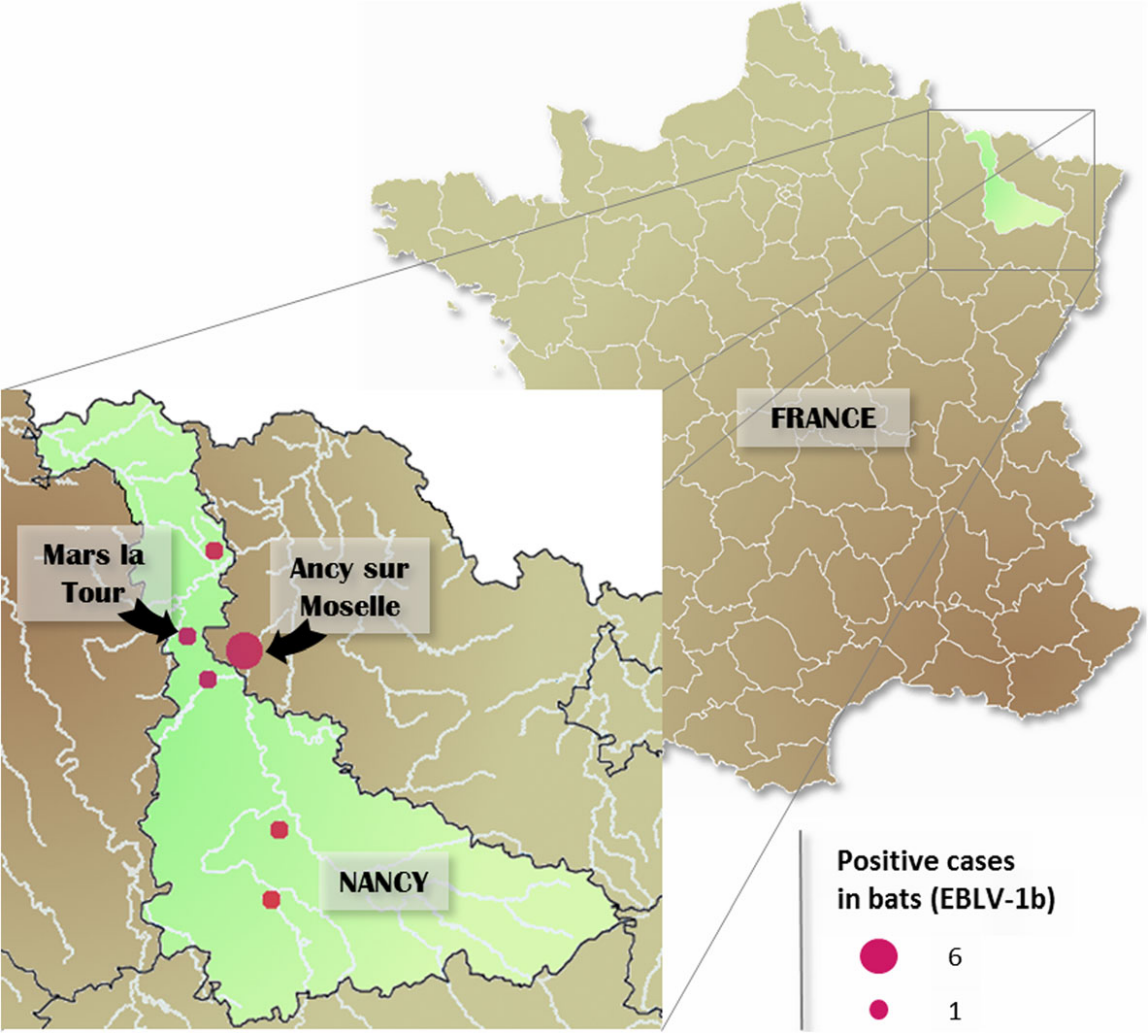
Map of France showing the location of the serotine bat colony in North-East France with the geographical distribution of cases of bat rabies reported in Moselle and Meurthe & Moselle from 1989 to 2016

An unusual mortality of juvenile serotine bats was reported in June 2009 in the centre of Ancy sur Moselle in the Moselle department in North-East France (lat. 49.054158, long. 6.058299) (Fig. 1). Approximately 30 to 40 juveniles were found dead in a large luxurious house in June of that year. The 30–40 juveniles were found in a maternity colony of serotine bats consisting out of ~200 pregnant female bats. The maternity colony, identified in attic of the private house since many years, was regularly observed by the owner. No case of unusual mortality was previously recorded in that colony. The 30–40 carcasses were not submitted for rabies diagnosis by the owner who alerted different services, including her veterinarian, the Mayor’s office and bat biologists. Three weeks after the report of the unusual mortality among serotines, nine bats were found dead in the attic by the owner. The nine cadavers were collected in the attic of the private house and sent for analysis to ANSES’s Nancy Laboratory for Rabies and Wildlife. The laboratory techniques used for routine diagnosis were the ones recommended by WHO and OIE [9, 10]: fluorescent antibody test (FAT) to detect antigens, rabies tissue culture infection test (RTCIT) to detect the infectious virus and in cases of positive rabies diagnosis an additional RT-PCR to detect viral RNA.

The Fluorescent Antibody Test was carried out on brain tissue specimens as described previously [10]. The RTCIT was used to confirm the presence of live virus, as previously described [9]. Viral RNA was extracted from 200 μL of supernatant from a 10% (*w*/*v*) brain suspension, then subjected to the partial Nucleoprotein (N) gene amplification, as previously described [11]. PCR products were bi-directionally sequenced by Beckman Coulter Genomics (Takeley, Essex, United Kingdom) with the same specific primers used in the nested PCR [11]. Sequence alignment of the six EBLV-1 sequences of Ancy sur Moselle and the EBLV-1 sequence of Mars la Tour was generated using the BioEdit software v. 7.2.5. based on the 600 bp region of the nucleoprotein gene.

Table 1 summarises the results of rabies infection in dead bats from the maternity colony and the results of passive surveillance undertaken in the two departments, Moselle and Meurthe et Moselle.

**Table 1:** Results of passive bat rabies surveillance undertaken in Moselle and Meurthe & Moselle

Studied zone	Area (km^2^)	Total found dead	Bats not analysed	FAT	RTCIT	hnRT-PCR	Typing of virus
Moselle	6216	28	12	6^a^ (10)	6 (10)	7 (9)	EBLV-1
Meurthe & Moselle	5246	12	2	1^b^ (9)	1 (9)	1 (9)	EBLV-1

Passive bat rabies surveillance was strengthened in Moselle and Meurthe & Moselle following the report of rabies infection in dead bats found in an *Eptesicus serotinus* maternity colony in Ancy sur Moselle

Analysis covered the period from 29 June to 15 October 2009

Of 28 bats found dead in Moselle, 19 were found dead in the maternity colony of Ancy sur Moselle. Ten were not analysed, six tested positive by FAT and three tested negative by FAT

Values in brackets correspond to the number of negative samples; the number of positive samples is shown in bold and italics

^a^ Samples from Ancy sur Moselle (lat. 49.054158, long. 6.058299)

^b^ Samples from Mars La Tour (lat. 49.098675, long. 5.887584)

Of the nine cadavers collected on 29 June 2009, four were not analysed due to the advanced decomposition of the carcasses. Of the five analysable specimens, four were tested positive for EBLV-1 by referenced rabies diagnostic methods.

### Strengthening of the passive bat rabies surveillance

Passive bat rabies surveillance was reinforced through a prefectural order in the departments of Moselle and Meurthe & Moselle following the report of rabies infection in the colony in Ancy sur Moselle [12]. Two additional positive cases were recorded in the maternity colony in two juvenile bats found dead respectively 7 and 11 days after the first four positive cases. Between July and October, a further eight cadavers were collected from the maternity colony. Two were tested negative and six were not analysed, due to the high level of decomposition of the carcasses. The colony’s mortality peak occurred between June and July, a further four serotine bats being found dead between August and October.

The strengthening of the passive surveillance scheme in the departments of Moselle and of Meurthe & Moselle led to a further nine and twelve dead bats being reported in the respective departments (Table 1). Sixteen days after the first four cases in Ancy sur Moselle, a third juvenile serotine was reported infected with EBLV-1 in Mars la Tour (lat. 49.098675, long. 5.887584), 14 km away. No maternity serotine colonies were found in the vicinity of Mars la Tour. A nucleotide comparison of the partial nucleoprotein gene sequence (570-nt) amplified from the seven EBLV-1 isolates showed a 100% nucleotide identity, suggesting that the seven isolates (MG334623) could be related to the same viral strain. BLAST analysis showed 100% of nucleotide identity between the consensus sequence of the 7 positive isolates and the sequence AY245833, isolated in 2000 in the North-East of France.

### Active bat rabies surveillance

To complement passive surveillance, six sessions of capture were held at nightfall between July and September 2009 after nocturnal counting of bats to evaluate the size of the maternity colony (Table 2). Each captured bat was marked with a lipped bat band positioned on the forearm and after sampling, all bats were immediately released at the capture’s site. Oropharyngeal swabs were taken from 111 individual bats to detect any viable rabies virus and lyssavirus RNA. Blood samples were taken from 94 bats to detect neutralising EBLV-1 antibodies [13]. Captures, handling and sampling were undertaken following authorisation from the French Ministry of the Environment [14].

**Table 2:** Detection of infectious particles (RTCIT), viral RNA (hnRT-PCR) and antibodies (mFAVNt) in serotine bats

				Detection in bats of:				
Dates of capture	Nb. counted bats^a^	Genus and age of bats		Viable virus	Viral RNA	EBLV-1 antibodies		
				RTCIT	hnRT-PCR	mFAVNt	% with AB	95% CI
7 July	135	A	F	0/36	1/36	12/29	45.5% (15/33)	28.5–63.4
			M	–	–	–		
		I	F	1/2	1/2	2/2		
			M	0/2	0/2	1/2		
10 July	81	A	F	0/33	2/33	18/30	59.5% (22/37)	42.1–75.2
			M	0/1	0/1	0/1		
		I	F	0/2	0/2	1/2		
			M	0/4	1/4	3/4		
23 July	30	A	F	0/8	0/8	4/7	55.5% (5/9)	21.2–86.3
			M	–		–		
		I	F	0/1	0/1	1/1		
			M	0/1	0/1	0/1		
4 August	30	A	F	0/10	0/10	1/6	14.3% (1/7)	0.4–57.9
			M	–	–	–		
		I	F	–	–	–		
			M	–	0/1	0/1		
20 August	21	A	F	0/4	0/4	0/3	0% (0/3)	0.0–70.8
			M	–	–	–		
		I	F	0/1	0/1	–		
			M	–	–	–		
10 September	10	A	F	0/5	0/5	3/5	60% (3/5)	14.7–94.7
			M	–	–	–		
		I	F	–	–	–		
			M	–	–	–		
Total		A		0/97	3/97	38/81	49% (44/94)	36.4–57.4
		I		1/14	2/14	8/13		

Six capture/release sessions were held following the report of rabies infection in the maternity colony between July to September 2009, to complement passive bat rabies surveillance

^a^Nocturnal countings of bats were performed 1 day before each capture to evaluate the colony’s size

Values correspond to the number of positive samples out of all the tested samples

*Abbreviations: A* Adult, *AB* antibody, *F* female, *I* Immature, *M* male

Oral swabs stored in 0.3 mL of culture medium were analysed using RTCIT on murine neuroblastoma cells [9] and by conventional RT-PCR [11]. To detect EBLV-1-specific neutralising antibodies in blood samples, a modified FAVN test was performed [13] with an EBLV-1 virus strain (ANSES, No. 121411) isolated in France in 2000. Samples were tested in quadruplicate, in threefold dilutions on BHK-21 cells with a starting dilution of 1/27. Controls included uninfected BHK-21 cells, OIE positive dog serum, negative dog serum and back-titration of the specific EBLV-1 virus. Levels of virus neutralizing antibodies were expressed in log D50. The threshold of antibody detection was calculated by using the Spearman–Karber formula and set at 1.67 log D50.

Table 2 summarizes the detection of infectious particles, viral RNA and antibodies in serotine bats. For each independent session, we computed the proportion of seropositive bats. Interval confidence of proportions (95% CI) were computed using the Bernoulli exact method. Results showed that one animal – a seropositive juvenile female captured only once— out of the 111 tested was found to shed viable virus in saliva, and five individuals were found positive for lyssavirus RNA. Viral RNA was detected during the first capture for two individual bats and during the second capture for three other individual bats (Table 2). Of the five animals found positive for the detection of RNA, three (2 females—one adult and one juvenile—and one juvenile male) were only captured once. Two animals were recaptured and sampled twice. One adult female was found positive during the first capture and surprisingly was found negative for the detection of RNA in the second capture. By opposite, the second animal —a female adult— was shown negative by RT-PCR in the first capture, and shown positive in the second one. The two animals were found to be seronegative for the detection of EBLV-1 antibodies. Not all animals from the colony developed a detectable neutralising antibody response, 49% of bats seroconverting between July and September with a detection threshold set at 1.67 logD50 [15]. Serology results showed an apparent decrease of the proportion of seropositive bats from July to September 2009, but the differences were not significantly different.

## Discussion and conclusions

We described here an unusual mortality among bats with six juveniles found dead in a short interval of time coupled to a high ratio of seropositive bats in a bat colony counting ~ 200 individuals. While the detection of EBLV-1 antibodies is frequently reported in field bat studies, the mortality of numerous bats from one site is unusually reported. The detection of viral RNA and viable virus in oropharyngeal swabs that we reported in the study is in accordance with previous studies [16]. Repeated detection of viral RNA in saliva swabs from recaptured bats was not observed in this study. These results are in accordance with the study of Vasquez-Moron et al. [17], that showed similar results in a serotine bat colony targeted after the detection of a bat rabies case. The majority of positive RT-PCR results are commonly associated with serotine bats. We showed here a high detection rate of neutralising EBLV-1 antibodies in the colony, with 49% of seroconverting bats. The detection of EBLV-1 virus-neutralizing antibodies is frequent in bats [16, 18], with a seroprevalence varying according to the site location, species and time. The high rate of seropositive bats in this study is probably indicative of an efficient virus transmission coupled to a rapid circulation of EBLV-1 in the colony.

**Fig. 2:**
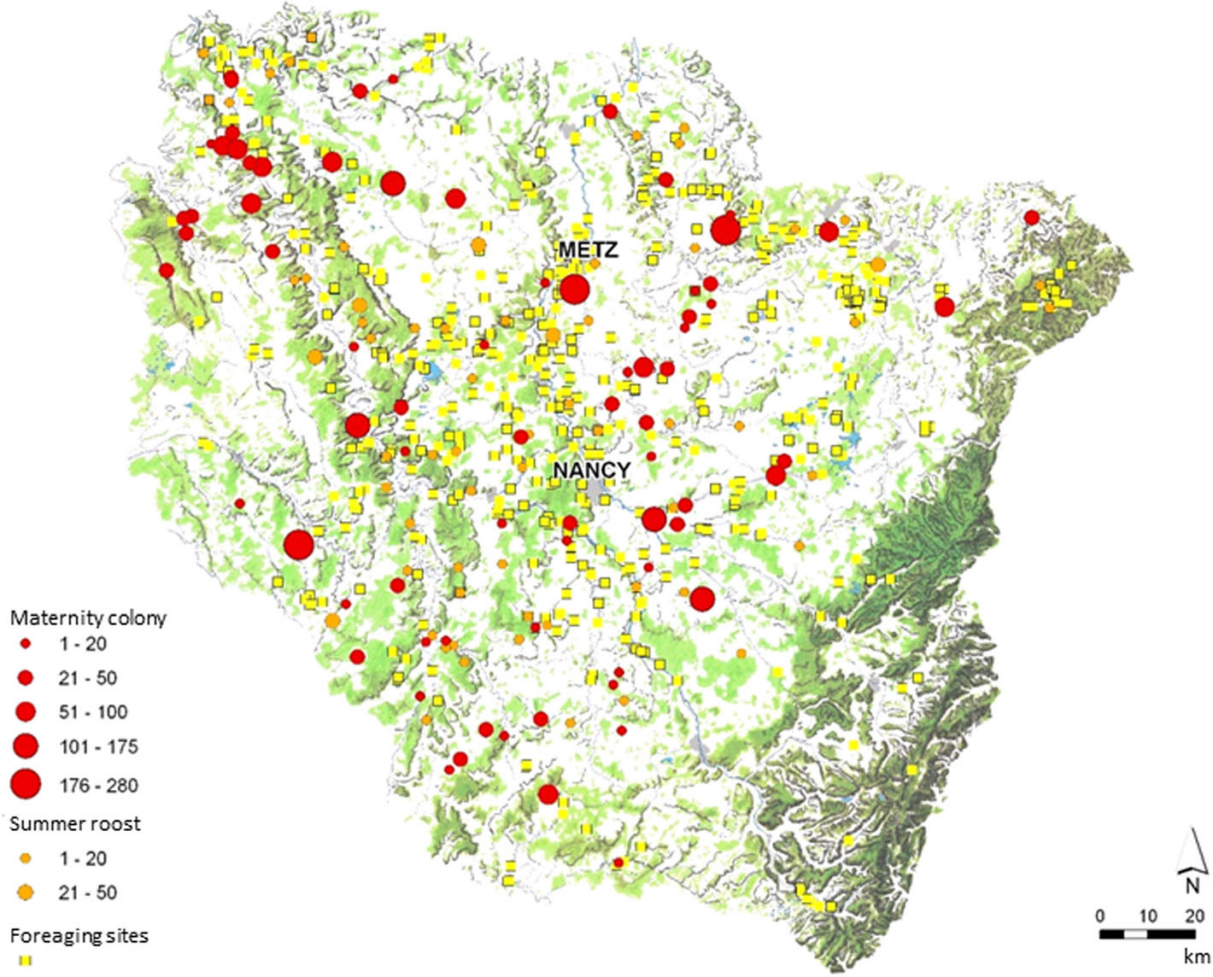
Map of the Lorraine region (area of 23,547 km^2^) showing the geographical distribution of serotine bats

Bats and their roosts are protected by French and European legislation. Bats play a key role in regulating insect populations and are one of the best natural indicators of the environment’s health. The serotine bat, considered as the reservoir of EBLV-1, is a common and widely distributed species in France generally and the Lorraine region particularly (Fig. 2) [19]. While some aspects of serotine bat ecology have been studied, including habitat use and roosting behaviour, little is known about the bats’ winter ecology, dispersal and seasonal connectivity with other species. It should be noted that three *Myotis emarginatus* bats were observed in August in the roost site. One of them was captured and subjected to lyssavirus analysis, but tested negative by FAT and PCR.

While bat rabies is a public health concern in Europe, the epidemiology and the pathogenicity of EBLV-1 in bats are still unknown as well as the dynamic of the infection and the virus influence on the mortality rate in bat colonies. To better understand the mechanisms by which EBLV(s) are maintained within bat populations, it is necessary to improve the passive surveillance of rabies in bats and further investigate serotine bat ecology through close collaboration between bat biologists and scientists. When possible, a colony of bats found to be naturally infected with EBLV-1 should be preserved and studied to shed light on the ecology of bat diseases. Neither human nor animal contact with the infected bat colony was identified. The owner of the house in Ancy sur Moselle received a post-exposure prophylaxis, although she did not report exposure. The house owner’s dog, already identified by microchip and vaccinated against rabies, received a booster vaccination in accordance with French regulations.

## Additional file

Additional file 1: Table S1. Results of passive bat rabies surveillance undertaken in Moselle and Meurthe & Moselle. Results are detailed by department, date, city of isolation and species of bats. (DOCX 23 kb)

## Abbreviations

BBLV: Bokeloh bat lyssavirus
EBLV-1: European bat 1 lyssavirus
EBLV-2: European bat 2 lyssavirus
FAT: Fluorescent antibody test
RNA: Ribonucleic acid
RTCIT: Rabies tissue culture infection test
RT-PCR: Reverse-transcription polymerase chain reaction
SFEPM: National bat conservation network
WCBV: West Caucasian bat lyssavirus
WHO: World Health Organisation
OIE: World Organisation for Animal Health

## References

[1] ICTV virus taxonomy: 2016 release (http://www.ictvonline.org/virustaxonomy.asp). Accessed 02 November 2017.

[2] Kuzmin I, Rupprecht C, Wang L, Cowled C (2015). Bat Lyssaviruses. Bats and viruses: a new frontier of emerging infectious diseases.

[3] Arechiga Ceballos N, Vazquez Moron S, Berciano JM, Nicolas O, Aznar Lopez C, Juste J, Rodriguez Nevado C, Aguilar Setien A, Echevarria JE: Novel lyssavirus in bat, Spain Emerg Infect Dis 2013; doi: 10.3201/eid1905.121071. 10.3201/eid1905.121071 PMC3647500 23648051

[4] Schatz J, Fooks AR, McElhinney L, Horton D, Echevarria J, Vazquez-Moron S, Kooi EA, Rasmussen TB, Muller T, Freuling CM. Bat rabies surveillance in Europe. Zoonoses Public Health. 2013; doi: 10.1111/zph.12002. 10.1111/zph.12002 22963584

[5] Freuling CM, Abendroth B, Beer M, Fischer M, Hanke D, Hoffmann B, Hoper D, Just F, Mettenleiter TC, Schatz J, Muller T: Molecular diagnostics for the detection of Bokeloh bat lyssavirus in a bat from Bavaria, Germany Virus Res 2013; doi: 10.1016/j.virusres.2013.07.021. 10.1016/j.virusres.2013.07.021 23932899

[6] Picard-Meyer E, Servat A, Robardet E, Moinet M, Borel C, Cliquet F. Isolation of Bokeloh bat lyssavirus in Myotis Nattereri in France. Arch Virol. 2013; doi: 10.1007/s00705-013-1747-y. 10.1007/s00705-013-1747-y 23760600

[7] Botvinkin AD, Poleschuk EM, Kuzmin IV, Borisova TI, Gazaryan SV, Yager P, et al. Novel lyssaviruses isolated from bats in Russia. Emerg Infect Dis 2003;9(12):1623-1625. Epub 2004/01/15. doi: 10.3201/eid0912.030374. PubMed PMID: 14720408; PubMed Central PMCID: PMC3034350. 10.3201/eid0912.030374 PMC3034350 14720408

[8] Picard-Meyer E, Robardet E, Arthur L, Larcher G, Harbusch C, Servat A, Cliquet F. Bat rabies in France: a 24-year retrospective epidemiological study. PLoS One. 2014; doi: 10.1371/journal.pone.0098622. 10.1371/journal.pone.0098622 PMC4044004 24892287

[9] Servat A, Picard-Meyer E, Robardet E, Muzniece Z, Must K, Cliquet F. Evaluation of a rapid Immunochromatographic diagnostic test for the detection of rabies from brain material of European mammals. Biologicals. 2012; doi: 10.1016/j.biologicals.2011.12.011. 10.1016/j.biologicals.2011.12.011 22245544

[10] Meslin F, Kaplan M, Koprowski H. Laboratory techniques in rabies. 4th ed. Geneva: World Health Organization. 1996.

[11] Picard-Meyer E, Bruyere V, Barrat J, Tissot E, Barrat MJ, Cliquet F: Development of a hemi-nested RT-PCR method for the specific determination of European Bat Lyssavirus 1. Comparison with other rabies diagnostic methods. Vaccine 2004; doi: 10.1016/j.vaccine.2003.11.015. 10.1016/j.vaccine.2003.11.015 15121304

[12] Treffel J-F. Arrêté de mise sous surveillance d'une population de chauves-souris (sérotines communes) d'où est susceptible d'être issue une chauve-souris porteuse d'un Lyssavirus. Arrété préfectoral, Préfecture de la région Lorraine 2009; 1-3.

[13] Cliquet F, Aubert M, Sagne L (1998). Development of a fluorescent antibody virus neutralisation test (FAVN test) for the quantitation of rabies-neutralising antibody. J Immunol Methods.

[14] Wintergest J. Dérogation ministérielle de capture, prélèvement et transport de chiroptères dans le cadre de la mission d'épidémiosurveillance et de recherche sur la rage des chiroptères 2012; 1–4.

[15] Picard-Meyer E, Dubourg-Savage MJ, Arthur L, Barataud M, Becu D, Bracco S, Borel C, Larcher G, Meme-Lafond B, Moinet M, et al. Active surveillance of bat rabies in France: a 5-year study (2004-2009). Vet Microbiol. 2011; doi: 10.1016/j.vetmic.2011.03.034. 10.1016/j.vetmic.2011.03.034 21570221

[16] Schatz J, Ohlendorf B, Busse P, Pelz G, Dolch D, Teubner J, Encarnacao JA, Muhle RU, Fischer M, Hoffmann B (2014). Twenty years of active bat rabies surveillance in Germany: a detailed analysis and future perspectives. Epidemiol Infect.

[17] Vazquez-Moron S, Juste J, Ibanez C, Ruiz-Villamor E, Avellon A, Vera M, Echevarria JE (2008). Endemic circulation of European bat lyssavirus type 1 in serotine bats, Spain. Emerg Infect Dis.

[18] Amengual B, Bourhy H, Lopez-Roig M, Serra-Cobo J (2007). Temporal dynamics of European bat Lyssavirus type 1 and survival of Myotis Myotis bats in natural colonies. PLoS One.

[19] Plan National d’Actions en faveur des chiroptères : 2009–2013: Bilan technique et financier des 5 ans du PNA 2009–2013, le diagnostic des 34 espèces, Sérotine commune, *Eptesicus serotinus*. http://www.plan-actions-chiropteres.fr/IMG/diagnostic-especes-chiropteres-2eme-PNA-FINAL.pdf (2017). Accessed 30 August 2017.

