# Supplementary material for: Bat rabies surveillance in France: first report of unusual mortality among serotine bats
Source: BMC Vet Res. 2017 Dec 13;13:387. doi: 10.1186/s12917-017-1303-1 (PMC5729292; doi:10.1186/s12917-017-1303-1)
Supplement: Additional file 1: Table S1. — Results of passive bat rabies surveillance undertaken in Moselle and Meurthe & Moselle. Results are detailed by department, date, city of isolation and species of bats. (DOCX 23 kb) [file 12917_2017_1303_MOESM1_ESM.docx]

Table S1: Results of passive bat rabies surveillance undertaken in Moselle and Meurthe & Moselle. Results are detailed by department, date, city of isolation and species of bats.

| Date of isolation | City of isolation | | | Species | Bats not analysed | FAT | RTCIT | hnRT-PCR |
| --- | --- | --- | --- | --- | --- | --- | --- | --- |
|  | City | | Lat./Long. |  |  |  |  |  |
| *Studied zone: Moselle (*6,216 km^2^) | | | | | | | | |
| 29 June | Ancy/Moselle | 49.054158, 6.058299 | | E. serotinus | 4 | ***4*** *(1)* | ***4*** *(1)* | ***5*** *(0)* |
| 01 July | Ancy/Moselle | 49.054158, 6.058299 | | E. serotinus | 0 | *(1)* | *(1)* | *(1)* |
| 02 July | Ancy/Moselle | 49.054158, 6.058299 | | E. serotinus | 1 | 0 | 0 | 0 |
| 06 July | Ancy/Moselle | 49.054158, 6.058299 | | E. serotinus | 2 | **1** | **1** | **1** |
| 10 July | Ancy/Moselle | 49.054158, 6.058299 | | E. serotinus | 0 | **1** | **1** | **1** |
| 20 July | Hayange | 49.329827,  6.062166 | | P. pipistrellus | 0 | (1) | (1) | (1) |
| 30 July | Blies-Ébersing | 49.124204,  7.144254 | | P. pipistrellus | 1 | 0 | 0 | 0 |
| 05 August | Ancy/Moselle | 49.054158, 6.058299 | | E. serotinus | 1 | 0 | 0 | 0 |
| 12 August | Metz | 49.119309,  6.175716 | | P. pipistrellus | 0 | (1) | (1) | (1) |
| 21 August | Ancy/Moselle | 49.054158, 6.058299 | | P. pipistrellus | 1 | (1) | (1) | (1) |
| 21 August | Marieulles | 49.005107,  6.100523 | | P. pipistrellus | 0 | (1) | (1) | (1) |
| 21 August | Rodemack | 49.469075,  6.236234 | | R. ferrumequinum | 1 | 0 | 0 | 0 |
| 21 August | Metz | 49.119309,  6.175716 | | P. pipistrellus | 0 | (1) | (1) | (1) |
| 21 August | Montigny-Les-Metz | 49.099068,  6.147807 | | P. pipistrellus | 0 | (1) | (1) | (1) |
| 29 September | Plaine-de-Walsch | 48.690375,  7.114962 | | M. bechsteinii | 0 | (1) | (1) | (1) |
| 29 September | Plaine-de-Walsch | 48.690375,  7.114962 | | P. auritus | 0 | (1) | (1) | (1) |
| 15 October | Ancy/Moselle | 49.054158, 6.058299 | | E. serotinus | 1 | 0 | 0 | 0 |
| *Studied zone:* Meurthe & Moselle (5,246 km^2^) | | | | | | | | |
| 06 July | Vandeléville | 48.42787,  5.994219 | | P. austriacus | 1 | 0 | 0 | 0 |
| 06 July | Damelevières | 48.556756,  6.386019 | | P. pipistrellus |  | (1) | (1) | (1) |
| 15 July | Mars-la-Tour | 49.098675,  5.887584 | | E. serotinus |  | **1** | **1** | **1** |
| 24 July | Nancy | 48.692054,  6.184417 | | P. pipistrellus |  | (1) | (1) | (1) |
| 11 August | Nancy | 48.692054,  6.184417 | | ND | 1 | 0 | 0 | 0 |
| 21 August | Heillecourt | 48.65233,  6.197997 | | N. leisleri |  | (1) | (1) | (1) |
| 03 September | Nancy | 48.692054,  6.184417 | | P. pipistrellus |  | (1) | (1) | (1) |
| 03 September | Foug | 48.691261,  5.786495 | | P. pipistrellus |  | (1) | (1) | (1) |
| 06 October | Heillecourt | 48.65233,  6.197997 | | N. leisleri |  | (1) | (1) | (1) |
| 06 October | Nancy | 48.692054,  6.184417 | | P. pipistrellus |  | (1) | (1) | (1) |
| 06 October | Tomblaine | 48.683482,  6.221307 | | P. pipistrellus |  | (1) | (1) | (1) |
| 06 November | Atton | 48.890533,  6.088963 | | M. mystacinus |  | (1) | (1) | (1) |

Values in brackets correspond to the number of negative samples; the number of positive samples is shown in bold and italics.

Analysis covered the period from 29 June to 06 November 2009.

Abbreviations: M. bechsteinii: Myotis bechsteinii ; M. mystacinus: Myotis mystacinus ; N. leisleri: Nyctalus leisleri ; ND: Not determined ; P. auritus: Plecotus auritus ; P. austriacus: Plecotus austriacus ; P. pipistrellus : Pipistrellus pipistrellus ; R. ferrumequinum : Rhinolophus ferrumequinum
